# Non-additivity of molecule-surface van der Waals potentials from force measurements

**DOI:** 10.1038/ncomms6568

**Published:** 2014-11-26

**Authors:** Christian Wagner, Norman Fournier, Victor G. Ruiz, Chen Li, Klaus Müllen, Michael Rohlfing, Alexandre Tkatchenko, Ruslan Temirov, F. Stefan Tautz

**Affiliations:** 1 Peter Grünberg Institut (PGI-3), Forschungszentrum Jülich, 52425 Jülich, Germany; 2 Jülich Aachen Research Alliance (JARA)-Fundamentals of Future Information Technology, 52425 Jülich, Germany; 3 Fritz-Haber-Institut der Max-Planck-Gesellschaft, Faradayweg 4-6, 14195 Berlin, Germany; 4 Max-Planck-Institut für Polymerforschung, Ackermannweg 10, 55128 Mainz, Germany; 5 Institut für Festkörpertheorie der Universität Münster, Wilhelm-Klemm-Straße 10, 48149 Münster, Germany

## Abstract

Van der Waals (vdW) forces act ubiquitously in condensed matter. Despite being weak on an atomic level, they substantially influence molecular and biological systems due to their long range and system-size scaling. The difficulty to isolate and measure vdW forces on a single-molecule level causes our present understanding to be strongly theory based. Here we show measurements of the attractive potential between differently sized organic molecules and a metal surface using an atomic force microscope. Our choice of molecules and the large molecule-surface separation cause this attraction to be purely of vdW type. The experiment allows testing the asymptotic vdW force law and its validity range. We find a superlinear growth of the vdW attraction with molecular size, originating from the increased deconfinement of electrons in the molecules. Because such non-additive vdW contributions are not accounted for in most first-principles or empirical calculations, we suggest further development in that direction.

## Abstract (web summary)

Van der Waals interactions are difficult to calculate at an atomistic level for moderate sized structures due to the many distinct atoms involved. Here, the authors measure the van der Waals force between an organic molecule and a metal surface, examining the non-additive part of these interactions.

## Graphical abstract

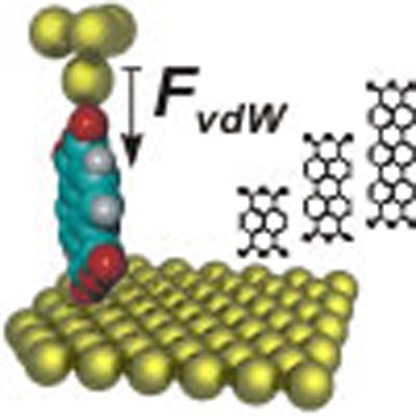

Even for two electrically neutral objects devoid of any static multipole moments, quantum mechanical fluctuations lead to the attractive dispersion or van der Waals (vdW) interaction^1^. The description of vdW forces as an inherently quantum mechanical phenomenon was developed for single atoms and homogeneous macroscopic bodies by London^1^, Casimir^2^ and Lifshitz^3^. For intermediate-sized objects like organic molecules an atomistic description is required, but explicit first-principles calculations are very difficult since correlations between many interacting electrons have to be considered^4^,^5^,^6^,^7^. The most accurate method available today to calculate electron correlations in general and the vdW interaction in particular, the coupled-cluster approach^8^, is computationally so expensive that it can only serve as a ‘gold-standard’ for small systems (up to ~100 light atoms). Hence, semi-empirical correction schemes for density functional theory (DFT) are often used that simplify the vdW interaction to a sum over atom-pair potentials^9^,^10^,^11^. Those dispersion correction schemes employ drastic simplifications: The vdW interaction is obtained by a pair-wise summation over atom–atom potentials and, related, the polarizability of complex objects such as multiatomic molecules is decomposed into a sum of atomic, possibly volume-scaled, polarizabilities. Moreover, the analytically derived asymptotic relation for the atom–atom potential 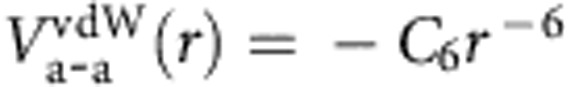 (refs 9, 10) is attenuated by a purely empirical damping function and used at short distances. In this situation, it is all the more unfortunate that a gap, similar to the one in theory, also exists between successful measurements of vdW and Casimir forces for single atoms on the one hand^12^,^13^,^14^,^15^,^16^ and macroscopic bodies on the other^17^,^18^, as comparable experiments for molecules are absent.

Here we present quantitative measurements of the vdW interaction that enable us to scrutinize the simplifications present in commonly used dispersion correction schemes and to reconstruct the asymptotic and short range vdW potentials in an approach that combines experiment and theory. We use the extremely sensitive force detection of an atomic force microscope^19^,^20^,^21^,^22^ in combination with its molecular manipulation capabilities^23^,^24^ to measure the distance dependence of the interaction between a series of related molecules and a Au(111) surface. The asymptotic atom-surface vdW potential 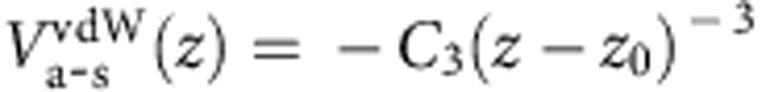 is closely related to the 1/*r*^−6^ atom–atom law and can be obtained from the latter analytically by integrating over half space^4^.

We have carried out our experiments on three *π*-conjugated poly-naphthalene derivatives, namely 1,4,5,8-naphthalene-tetracarboxylic dianhydride (NTCDA), and its perylene and terrylene counterparts PTCDA and TTCDA (Fig. 1). The investigation of this series of structurally related molecules allows us to gain insight into size-dependent effects. In contrast to previous force–distance measurements^17^,^18^ that were designed to determine vdW and Casimir interactions between macroscopic bodies, we perform force gradient (d*F*_*z*_/d*z*) versus distance (*z*) measurements, using a commercial qPlus quartz tuning fork sensor^19^ in a combined CREATEC scanning tunnelling/non-contact atomic force microscope^23^,^24^. It was recently demonstrated that qPlus sensors yield very precise force gradient spectra and images^20^,^21^,^22^.

In the experiment, the gold-covered tip of the qPlus sensor is approached at zero bias voltage to an isolated adsorbed molecule until a chemical bond between the tip and the molecule forms^23^,^24^,^25^,^26^,^27^,^28^. Then, the tip is retracted again, such that the contacted molecule is gradually lifted into an upright position^23^,^24^, detached from the surface and finally lifted by ~2 nm (Fig. 1). The molecule-surface attraction aligns the diagonal of the tip-suspended molecule with the surface normal. Throughout the lifting process, changes in the qPlus resonance frequency are recorded. Our qPlus sensor converts force gradients d*F*_*z*_/d*z* into resonance frequency shifts Δ*f* with a proportionality constant of *ξ*=8.4 Hz/(N m^−1^). The high stiffness of the qPlus sensor of 1,800 N m^−1^ and its small oscillation amplitude *A*<0.2 Å allow removing the molecules from the surface without abrupt rupture events. Thus, the manipulation is reversible and we perform up to 45 up-and-down cycles (90 Δ*f*(*z*) spectra) within one contacting experiment before the molecule is released from the tip by a voltage pulse. The contribution of tip-surface forces to the measured force gradient is removed by subtracting the bare-tip approach curve recorded at the beginning of each contacting experiment. The cleanliness and stability necessary for a straightforward data interpretation is achieved by working in ultra-high vacuum and at low temperature (*T*=5 *K*).

With our experiments, we are able to determine long-range molecule-surface vdW potentials in excellent agreement with theory. The results further allow us to confirm the asymptotic *r*^−3^ force law, to specify its validity range, and even to quantify the non-additive part of the vdW interaction, which is particularly challenging for theory. In the present case, experiments indicate that cooperative effects due to deconfinement of electrons^29^ account for approximately 10% of the total interaction. This non-additivity is of general validity in molecules and thus relevant at the intersection of chemistry, physics, biology and materials science^30^,^31^,^32^. As non-additive contributions (which can amount to several eV in biomolecules) cannot, by construction, be accounted for in state-of-the-art density functional calculations, we suggest further development in that direction.

## Results

### Force gradient measurements

Regarding the accuracy of the force gradient measurements, a peak-to-peak noise in Δ*f* below 0.05 Hz is required, more than one order of magnitude lower than in the seminal experiment in ref. 20. This is achieved by averaging over several hundred carefully aligned individual Δ*f* curves (each with ~0.4 Hz noise level), obtained in 11 NTCDA, 7 PTCDA and 7 TTCDA contacting experiments (see Methods section). These global averages exhibit a noise level as low as 0.02 Hz and form the basis of our analysis. The averaged curves from each individual contacting experiment are shown in Fig. 2a. We note that while the curves scatter considerably as long as the molecules are under the influence of the surface corrugation, they become perfectly reproducible (exemplified for TTCDA in the inset of Fig. 2a) in the region of interest where the molecules and the surface are well separated and the asymptotic vdW force law is expected to apply. It is this reproducibility that allows us to average over several contacting experiments.

### Tip height calibration

When determining a force gradient law d*F*_*z*_/d*z*(*z*), the accurate quantification of *z* is as important as the precise measurement of d*F*_*z*_/d*z*. As the orientation of the molecule is stabilized by the molecule-surface attraction, only the absolute height remains to be determined. Here we use the following solution: we use the lifted molecule itself as a ruler, employing the model of the lifting process that was developed in ref. 24. In short, the force gradients d*F*_*z*_/d*z*(*z*) measured when lifting the molecules from the flat into the upright configuration, together with molecular mechanics simulations and the known lengths of the molecules, allow us to precisely link relative experimental tip heights (*z*-piezo voltages) to absolute heights of the molecules above the surface. In that way, the molecular geometries, that is, individual atom heights *z*_*i*_ above the surface can be obtained for the entire lifting process. With a tip-molecule bond length of 2.2 Å we obtain tip-sample distances of *z*_tip_=13.4 Å (NTCDA), 17.5 Å (PTCDA) and 21.7 Å (TTCDA), for the upright molecules. The insets in Fig. 2a show the respective geometries.

### Fitting model

**Figure 1:**
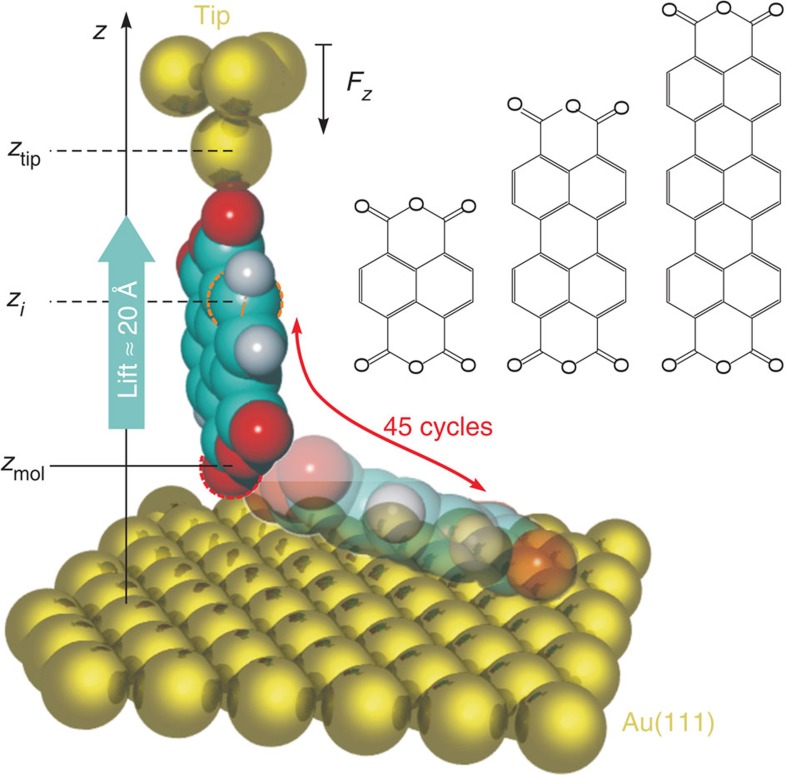
Schematic sketch illustrating the experiment. Within each experiment a single, isolated molecule is contacted at one of the reactive carboxylic oxygen atoms, detached from the surface, lifted about 2 nm further and brought back into the adsorbed state. While repeating this cycle up to 45 times, the force gradient d*F*_*z*_/d*z* is constantly recorded. The tip-surface and molecule-surface distances *z*_tip_ and *z*_mol_, as well as the distance *z*_*i*_ between each atom *i* and the surface are indicated. The chemical structures of the three investigated molecules are shown on the right (from left to right: NTCDA, PTCDA and TTCDA).

**Figure 2:**
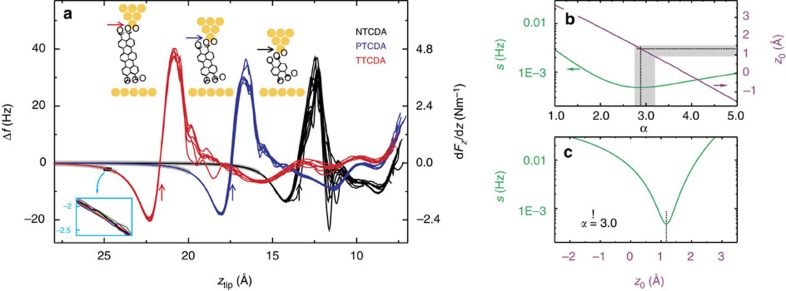
Determination of the force law. (**a**) Averaged frequency shift Δ*f* curves from individual contacting experiments. The Δ*f* on tip approach has been subtracted from the curves (see Supplementary Fig. 4). All curves are *z*-aligned at large *z*_tip_ where molecule and surface are well separated. The absolute *z*-scale is obtained by comparison to simulations^24^ (see Supplementary Fig. 5). The arrows indicate where each molecule is detached from the surface with cartoons at the top showing the respective geometries. The inset exemplifies reproducibility and noise level for TTCDA. The grey background marks the part of each curve that is used in the fit. (**b**) Fit quality *s* and fit parameter *z*_0_ for different force law exponents *α*. The best fit is obtained for *z*_0_=1.3 Å at *α*=2.85 (dotted lines). The corridor of physically reasonable *z*_0_ values (and of the respective exponents α) is shaded grey. (**c**) Fixing the exponent to *α*=3, we obtain a sharp minimum in *s* at *z*_0_=*d*_Au(111)_/2 (dotted line).

Theory predicts the asymptotic interaction potential for an atom in front of a surface to be a power law of the form


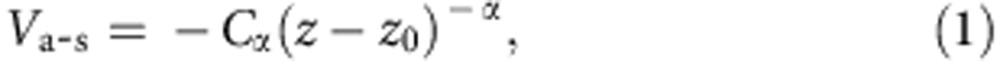


with material-specific coefficients *C*_*α*_ (depending on the atomic polarizability and the dielectric function of the substrate), the so-called vdW reference plane *z*_0_ (ref. 4), and the exponent *α* which for the dipolar dispersion interaction is 3 (see Methods section). Since in our experiment the tip-suspended NTCDA, PTCDA and TTCDA molecules are not coplanar with the surface and hence their vertical extensions in *z*-direction are similar to the molecule-surface separations (see Fig. 1), each molecule cannot be approximated (different from an atom) as a point object. Thus, the measured force gradient curves in Fig. 2a cannot be fitted directly with the second derivative of equation (1) because there is neither an unique value for *z* nor for *C*_3_. To be able to analyse our results in terms of the vdW force law, we therefore choose a representation commonly used in theory and formally represent the molecule by a collection of (fluctuating) atomic point dipoles. Corresponding to equation (1), the molecule-surface vdW potential


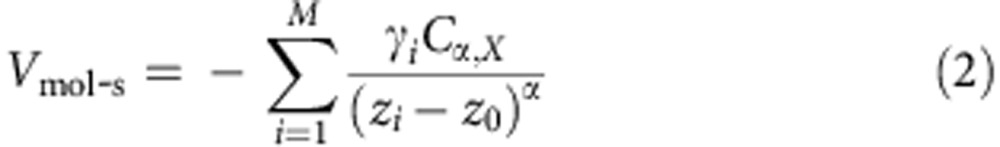


is then obtained by summing over *M* atoms at the heights *z*_*i*_ (Fig. 1) in each molecule. Since the atomic vdW coefficients defined by this approach, *C*_*α*,X_ with X=N(TCDA), P(TCDA) or T(TCDA) can be different for NTCDA, PTCDA and TTCDA, we go beyond the approximation of additive polarizabilities and vdW potentials, despite formally breaking the molecules up into atomic point dipoles. The reference plane *z*_0_, being a property of the surface, is identical for all three molecules. The distribution of polarizability within each molecule is estimated with the help of theory. We employ weighting factors *γ*_*i*_ that are taken from the semi-empirical dispersion correction scheme vdW^surf^ (ref. 11) (0.29 for hydrogen, 0.67 for oxygen and 1.0 for carbon, see Methods section and Supplementary Table 1). For a meaningful comparison with vdW^surf^, this choice (well defined but not unique in the theoretical framework) is preferable. Note that the precise choice of the *γ*_*i*_ has no influence on any of our conclusions.

In fitting our force gradient data with the second derivative 
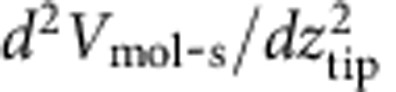
 of equation (2), we proceed in two steps. Initially, we examine the exponent *α* of the asymptotic behaviour found in experiment. Having established the exponent *α*=3, we then determine the *C*_3,X_ coefficients from equation (2) and analyse the result.

### Force law

To determine the force law, we vary the exponent *α* from 1 to 5 while optimizing *z*_0_ and all three *C*_*α*,X_ at each step. We use a weighted least-squares regression minimizing *s*(*z*_0_,*C*_*α*,X_), which is a measure for the goodness of the simultaneous fit of all three Δ*f* curves. More information on data processing and fitting can be found in the Methods section. The results of the fit are shown in Fig. 2b. The best fit is obtained for *α*=2.85, which is close to the expected value *α*=3 for the asymptotic vdW potential. Furthermore, there is a clear variation of *z*_0_ with *α*. In particular, the region of physically sensible values^4^ close to *z*_0_≈*d*_Au(111)_/2≃1.2 Å, where *d*_Au(111)_ is the Au(111) interlayer spacing, is quite narrow with 2.75<*α*<3.2. Both results confirm the asymptotic interaction between constituent parts of the molecules (‘atoms’) and the surface to be


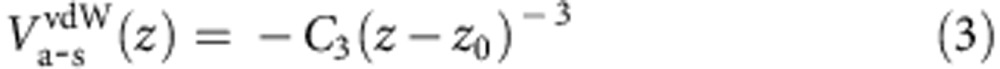


Further analysis shows that this expression is valid for *z*>4.8 Å (see below). This power law proves that in our experiment the vdW interaction dominates the molecule-surface potential, with other interactions playing a minor role. This conclusion is confirmed by a detailed analysis of electrostatic forces in our experiments (see Discussion).

As an important benchmark for theory, we find a sharp minimum in *s* precisely at the theoretically well-founded vdW reference plane position *z*_0_=*d*_Au(111)_/2 if we fix *α* to the asymptotic value of 3 (Fig. 2c).

### *C*_3_ coefficients

**Figure 3:**
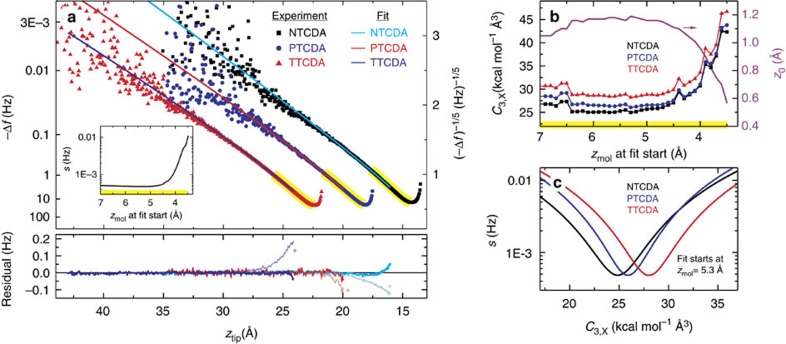
Determination of the asymptotic vdW coefficients. (**a**) Plot of the experimental Δ*f* data for the detached molecules (on a (−Δ*f*)^−1/5^ scale). The starting point of the fit intervals was varied within the yellow-marked regions that correspond to molecular heights 3.5 Å<*z*_mol_<7.0 Å. The displayed fits to the experimental data (solid lines) have been obtained for a starting point of *z*_mol_=5.3 Å. The inset shows how the fit quality *s* depends on the starting point of the fit interval. We show the residuals of each fit and compare them with the residuals (marked by an asterisk) obtained in a fit where all *C*_3,X_ are constrained to be identical, that is, without superlinearity. (**b**) Best-fit parameter values *z*_0_, *C*_3,N_, *C*_3,P_ and *C*_3,T_ as a function of the fit interval starting point. We obtain unphysical values if the asymptotic force law of equation (3) is used too close to the surface. (**c**) Dependency of the fit quality *s* on the change of a single *C*_3,X_ parameter. The three other fit parameters remain at their optimal values from **b**. The start of the fit interval was set to *z*_mol_=5.3 Å.

**Figure 4:**
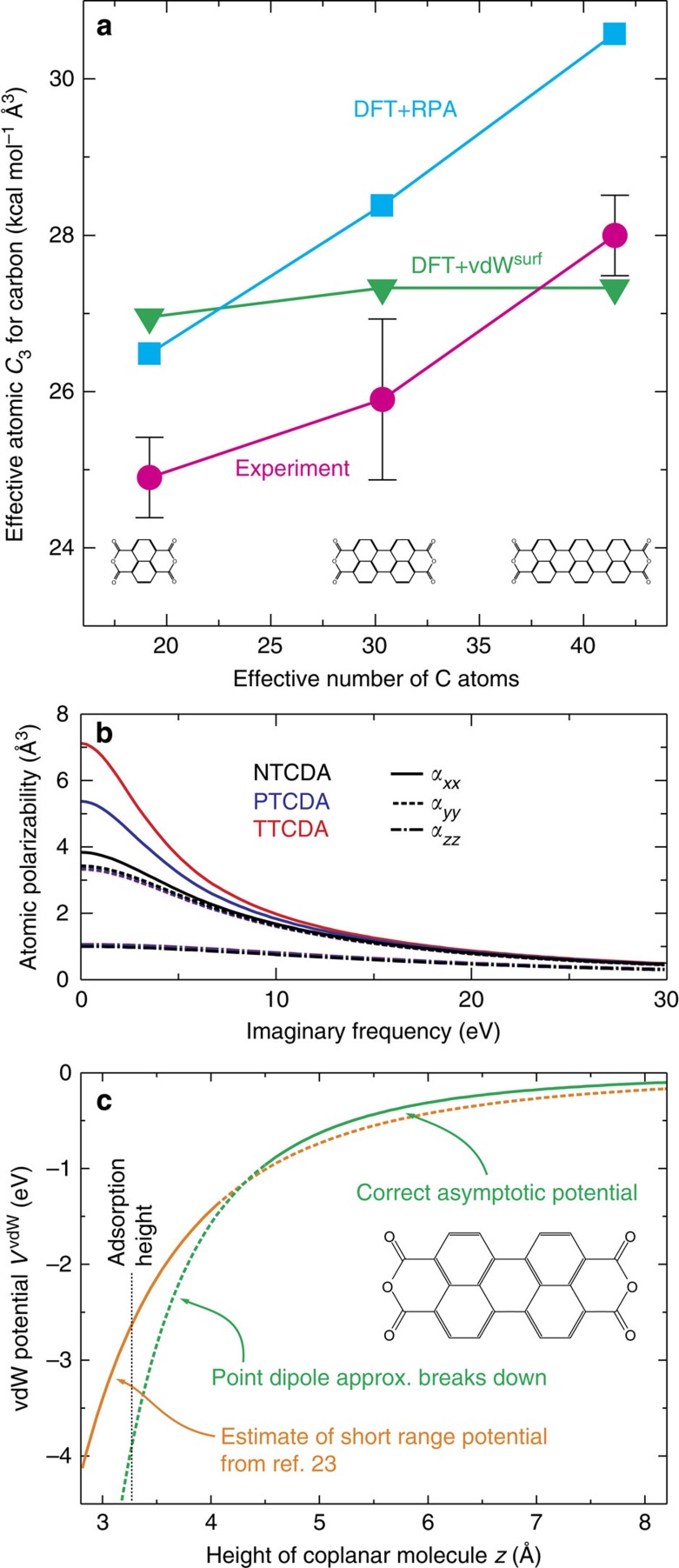
Experimental results and comparison with theory. (**a**) Summary of the experimentally obtained *C*_3_ values. The experimental error bars indicate the uncertainty in the *C*_3_ coefficients due to the influence of the experimental noise on the fitting routine. Calculated values from the semi-empirical dispersion correction scheme vdW^surf^ and from DFT+RPA are also shown. (**b**) Dynamic per-atom polarizabilities of carbon for NTCDA, PTCDA and TTCDA as resulting from RPA calculations. The coordinates *x*, *y* and *z* refer to the directions along the long axis, short axis and perpendicular to the plane of each molecule. (**c**) The true vdW potential of PTCDA (coplanar to the surface) deviates from the asymptotic form (green) at small molecule-surface separations (compare Fig. 3b). The potential close to the adsorption height is estimated by the orange curve calculated on the basis of ref. 24, while the true vdW potential is an interpolation between the two branches. The invalid parts of both potentials are dashed.

We now turn to the determination of precise *C*_3_ coefficients within the theoretical model given by equation (2). For a correct recovery of the (by definition asymptotic) *C*_3_ values, it is crucial to exclude the *z*_tip_-interval where the height *z*_mol_ of the lower end of the molecule above the surface (Fig. 1) is small and deviations from equation (3) are expected, due to Pauli repulsion, higher-order terms of the vdW multipole expansion, and the invalid point dipole approximation. To identify the minimal allowed *z*_mol_, we fit the experiments in intervals that start between *z*_mol_=3.5 and 7.0 Å (yellow regions in Fig. 3a) and end at the largest *z*_tip_ values reached. We find that all fit parameters (Fig. 3b) and the fit quality *s* (inset of Fig. 3a) converge to a plateau for *z*_mol_≥4.8 Å. Below this threshold, the fitted parameters depend strongly on the starting value of the fit region, with *z*_0_ becoming unphysically small. The value of the threshold is consistent with calculations in the random phase approximation (RPA)^33^ (see Supplementary Methods). Fits for a starting value of *z*_mol_=5.3 Å are displayed in Fig. 3a, while Fig. 3c shows how the fit quality depends on the individual *C*_3,X_ values. For all three molecules, we find a clear minimum in *s*, for NTCDA at *C*_3,N_=24.9 kcal mol^−1^ Å^3^, for PTCDA *C*_3,P_=25.9 kcal mol^−1^ Å^3^ and for TTCDA *C*_3,T_=28.0 kcal mol^−1^ Å^3^. The respective data points are plotted in Fig. 4a.

### Non-additivity of experimentally determined *C*_3_ coefficients

The *C*_3_ coefficients determined with our approach show a clear trend of increasing with molecular size, that is, the per-atom molecule-surface interaction rises in the sequence NTCDA, PTCDA and TTCDA. This is a clear signature of cooperative effects between the atoms in the extended *π*-electron system, confirming the importance of the non-additivity in the molecular polarizabilities without which our experimental findings cannot be explained. This superlinearity accounts for ~10% of *C*_3_ for TTCDA if compared with NTCDA.

Figure 4a also displays *C*_3_ coefficients from the computationally expensive DFT+RPA method. It calculates the macroscopic response of the molecule to electrical fields from a full microscopic quantum theory of the molecule, allowing insight into the role played by the quantum mechanical electronic states. Apart from slightly larger absolute values, it predicts a superlinearity in good agreement with experiment. The origin of the superlinear rise of *C*_3_ is the increasing deconfinement of electrons in the direction of the long molecule axis. This leads to a strong increase in the per-atom polarizability of the carbon atoms (averaged over all carbon atoms in each molecule) for NTCDA, PTCDA and TTCDA at small imaginary frequencies along the respective axis (Fig. 4b). The anisotropy originates from the anisotropic shape of the molecules. For an infinitely long molecule, electrons would form a metallic band and the static polarizability in this direction would diverge. For finite molecules, the band breaks up into confined states, which are the molecular orbitals^34^. The longer the molecule, the more closely spaced are these confined states on the energy axis. This leads to increasing contributions of corresponding electronic transitions to the low-frequency molecular polarizability and hence to *C*_3_. Indeed, we find that the rising weight of the transition between the highest occupied and lowest unoccupied orbitals alone is responsible for more than 90% of the superlinearity from NTCDA to TTCDA (see Supplementary Fig. 9). In essence, the deconfinement of valence electrons washes out the ‘atomic individuality’ in the molecule and introduces cooperative behaviour. More details can be found in the Supplementary Discussion.

By construction, the semi-empirical dispersion correction vdW^surf^ does not exhibit a superlinear increase of *C*_3_ (Fig. 4a), because it is based on volume-scaled atomic polarizabilities (see Supplementary Methods). However, the fact that its deviation from the experiment is of similar size as the experimentally observed superlinearity suggests that with vdW^surf^ a semi-empirical correction scheme of sufficient accuracy is available such that efforts to include cooperative effects would make sense. We stress that this would constitute a most important advance, because for systems involving larger molecules, such as functional self-assembled monolayers or surface-immobilized biomolecules, 10% of the total vdW interaction, which may amount to several eV, are a significant energy that can influence the properties profoundly. The conclusions drawn here are valid not only for dispersion corrected DFT methods but for the entire class of force-field based simulations of conjugated molecules.

### Surface holding potential

We briefly note here that in conjunction with the analysis of ref. 24, the results of the present work mean that the complete adsorption potential of a large organic molecule has been mapped out by force experiments, including the asymptotic (this paper) and the short-distance^24^ regimes (see Supplementary Discussion). The asymptotic potential is plotted in Fig. 4c (green). We have reported here that for *z*<4.8 Å, the asymptotic potential loses its validity. In this region, the potential extracted from force measurements with the molecule close to the surface (orange)^24^ is a good approximation.

## Discussion

We end the paper by discussing two possible sources of systematic errors. First, the presence of electrostatic forces in the junction; since these are also long ranged, they could add to measured force gradients. Second, a deviation of the orientation of the molecule in the junction from the vertical; this could invalidate the atomic positions that enter our fitting procedure. Our analysis shows that both electrostatic forces and deviations from the vertical orientation do not play a significant role.

While it is generally true that precise positions of molecules in scanning probe junctions are difficult to establish, our experiment offers an exceptional degree of control: Since we attach the tip to the molecule while it is still flat on the surface and then retract the tip gradually, we can be sure that at the point when the molecule leaves the surface it does so in the upright geometry. The force gradient data prove this unambiguously. The crucial question is whether this geometry is maintained when the tip is retracted further. To analyse this question, we have determined the directionality of the tip-oxygen bond, since a significant directionality of this bond would be one mechanism that could tilt the molecule out of the vertical once the contact to the substrate is broken. A DFT calculation (see Supplementary Discussion) shows that there is essentially no directionality of this bond in a broad angular range. While this means that the tip-oxygen bond will not rotate the molecule out of the vertical, it also implies that it will not pull it back into the vertical if for some other reason it tilts out of the vertical. But as we will argue now, the absence of a restoring force from a directional bond means that any tilting is easily detectable in experiment (and indeed sometimes detected).

Hypothetical asymmetries in the tip would induce a torque on the tip-suspended molecule. If the molecule responds to this torque by rotating towards the tip, the torque increases further and finally flips the molecule completely to the tip (see Supplementary Discussion and Supplementary Figs 7 and 8). Experiments where we observe this effect have been excluded from our analysis (see Methods section and Supplementary Figs 1–3). If the molecule does not flip up, the torque must be so small that even at the largest tip-surface distances in the experiment the restoring force originating from the molecule-surface attraction stabilizes the molecule in the vertical orientation. Since those restoring forces increase rapidly as *z*^−4^ when approaching the surface (whereas the tilting torque arising from the tip asymmetry is independent of the tip-surface distance and thus remains small), they completely dominate any tip-induced torque in the relevant part of the experiment. The molecular orientation is thus determined by the molecule-surface interaction only, as in our molecular mechanics model that yields the employed *z*_*i*_, with the result that the orientation of the molecule is vertical.

Regarding the electrostatic forces, we have calculated the electrostatic interaction energy between the three tip-attached molecules and the surface for an exemplary molecular height of *z*_mol_=7 Å. We find that this interaction is only a few meV for all three molecules (−3.1 meV for NTCDA, −5.1 meV for PTCDA and −2.1 meV for TTCDA, see Supplementary Discussion). At this distance, the vdW interaction energy as calculated with vdW^surf^ is approximately one order of magnitude larger. The reason for the very small electrostatic interaction is the fact that there is no large-scale charge transfer between tip and molecule; all bond-related charge reorganization takes place in the direct vicinity of the local tip-oxygen bond. Hence, electrostatic forces are not expected to influence the measured frequency shift curves, in full agreement with the observed force law exponent *α*=3.

To conclude, we have employed the extremely sensitive force detection of an atomic force microscope and measured the long-range vdW potentials between a series of related molecules and a metal surface. In particular, the exponent of the force law, the reference plane position *z*_0_, the validity range of the asymptotic force law, the absolute values of the *C*_3_ coefficients and their superlinearity have been determined, all in excellent agreement with theory. An analysis of the mechanical and electronic properties of the bonding between the *X*TCDA and an Au-covered tip has shown that crucial properties of this material system, which allow the quantitative determination of vdW forces between a single molecule and a surface, even up to the possibility to record the superlinearity of vdW forces, are on one hand the almost complete absence of a directionality of the bond between the functional oxygen atom and the tip, and on the other hand the absence of any significant charge transfer between tip and molecule.

## Methods

### Sample and tip preparation

The Au(111) single crystal is cleaned using the standard routine of Ar-sputtering and annealing. NTCDA, PTCDA and TTCDA are deposited at sub-monolayer coverage onto the room-temperature sample by thermal evaporation at 500, 570 and 710 K, respectively. After deposition, the PTCDA and TTCDA samples are annealed to 470 K for 2 min. Single molecules are created in the scanning tunnelling/non-contact atomic force microscope by detaching them from the edge of an island with the tip and dragging them several nm away. The tip of the qPlus sensor is made from a PtIr wire of 15 μm diameter that is cut and sharpened by a focused ion beam. The tip apex is covered with gold^26^. This is achieved by carefully dipping it into the Au surface.

### Treatment and averaging of experimental raw data

The experimental raw data has been measured in 25 individual contacting experiments (11 on NTCDA, 7 on PTCDA and 7 on TTCDA). Each of these consists of (1) a vertical approach of the bare Au-covered tip towards the position of one of the carboxylic oxygen atoms within a single isolated surface-adsorbed molecule, (2) the jump to contact at which the carboxylic oxygen atom flips up and forms a covalent bond with the tip and (3) a series of up to 45 vertical tip retraction and reapproach cycles with the molecule attached to the tip. In all phases, the frequency shift of the qPlus sensor is recorded as a function of z-piezo voltage (relative *z*-coordinate *z*_rel_).

Irregular curves, which arise as a consequence of either a flip-up of the entire molecule to the tip, a broken tip-molecule bond or an instability of the tip-suspended molecule are removed from the data set (about 15% (NTCDA), 20% (PTCDA) and 50% (TTCDA) of all curves). An instability of the suspended molecule is characterized by a pronounced hysteresis between the Δ*f*(*z*) curves taken during retracting and approaching of the tip (see Supplementary Fig. 2). We show exemplary raw data curves in the Supplementary Figs 1–3 to illustrate our criteria for curve-removal.

Within each contacting experiment, the remaining individual Δ*f*(*z*_rel_) curves are aligned on the *z*_rel_-axis with the first reapproach curve, focusing on the part of each curve where the molecule is well separated from the surface. The first reapproach curve is chosen because (1) it is measured in the same direction (tip lowering) as the bare-tip approach curve and hence has the same (small) PLL lag and (2) it is measured shortly after the bare-tip approach curve and hence is barely affected by any slow *z*-piezo creep that may occur. The aligned Δ*f*(*z*_rel_) curves are averaged. This greatly reduces the noise level.

Next, a fit to the bare-tip approach curve Δ*f*_0_(*z*_rel_) is subtracted from the average curve. This step eliminates the tip-surface interaction from the data. Note that the contribution of the single (still flat adsorbed) molecule to the approach curve is too small to be relevant since, at identical tip heights, the tip-suspended vertical molecule is always much closer to the surface than the flat molecule was to the bare tip. This is the reason why the flat molecule’s contribution to the bare-tip approach curve can be neglected.

Since the noise level of the bare-tip approach curve is that of an individual Δ*f*(*z*_rel_) curve (±0.4 Hz) and thus much higher than the noise of the average curve, we subtract a fit to the bare-tip approach curve instead of the bare-tip approach curve itself. The fit is performed using the nine-parameter function


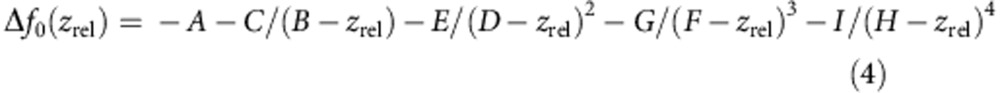


An example of such a fit is shown in Supplementary Fig. 4.

After subtracting the fitted bare-tip approach curve, the (averaged) Δ*f*(*z*_rel_) curves of each contacting experiment for one molecular species are aligned on the *z*_rel_-axis and averaged again. This further reduces the noise level. Finally, the three resulting curves, one for each molecular species, are aligned with respective simulations of the full single-molecule manipulation process that are performed using the procedure and parameters described in ref. 24 (see Supplementary Fig. 5). This final step provides us with the correctly calibrated *z*_tip_-axis. The simulations allow us to employ the known lengths of the molecules as highly accurate rulers for the determination of the absolute tip-sample distance *z*_tip_ in the experiments.

### Multipole expansion for the dispersion interaction

A correct description of molecule-surface interaction requires the inclusion of both exchange and correlation at a consistent level. Exchange is the part of the electron–electron interaction energy (beyond the Hartree term) that is related to the antisymmetric nature of the many-electron wave function^4^, while correlation is the correction to the total energy in the Hartree–Fock approximation^4^. If the distance *z* between the adsorbate and the substrate is large and there is thus no wave function overlap, there will be no exchange. In this limit, correlation can be treated perturbatively and Lifshitz–Zaremba–Kohn theory results in an asymptotic power series −*C*_3_/*z*^3^−*C*_4_/*z*^4^−*C*_5_/*z*^5^−… (ref. 35), where *C*_3_ depends on the dipole polarizability of the adsorbate and the bulk macroscopic dielectric function of the metal in the long wavelength *q*=0 limit (leading order dispersion interaction). Higher-order terms include combinations of multipole adsorbate polarizabilities and *q*-dependent substrate response. In a common approximation, we account for the first two terms of this series by


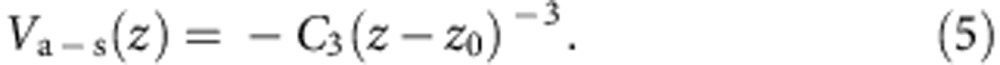


The *C*_3_ coefficient in equation (5) is given by ref. 4


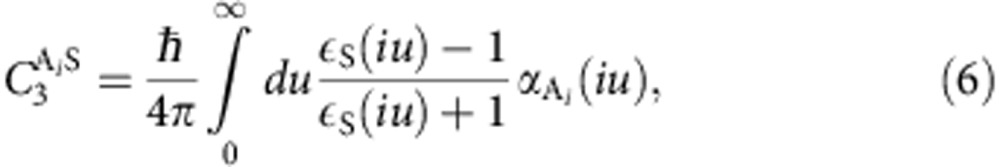


with *ε*_S_(*iu*) being the dielectric function of the metal and *α*_A*i*_(*iu*) the atomic polarizability of species *A*_*i*_. *z*_0_=*C*_4_/3*C*_3_ gives the position of the vdW reference plane that is closely related to the dynamic image plane of the surface^4^. Usually, *z*_0_ lies within 20–30% of *d*_*hkl*_/2, where *d*_*hkl*_ is the distance between *hkl* lattice planes of the *hkl* substrate surface.

### Fit function

The experimental Δ*f*(*z*_tip_) curves are fitted by the second derivative of equation (2). The heights *z*_*i*_ of each atom *i* above the gold surface is taken from the simulation results shown in Supplementary Fig. 5. In the region of interest, where molecule and surface are well separated, all atoms in the molecule rigidly move up together with the tip, that is, the *z*_*i*_ increase collectively with the same rate as the experimental tip height is changed.

A clear and direct experimental evidence for a stable molecular configuration of the freely suspended molecule is the lack of any hysteresis between up- and down-cycles (that is, lifting and lowering of the molecule). If the molecule changed its configuration on the tip during the up-cycle, one would expect the down-cycle to show a different frequency shift curve compared to the up-cycle. Occasionally, this effect was observed (see Supplementary Fig. 2 and the discussion of data treatment above). Those curves were excluded from the data set before averaging and fitting.

Since the dynamic atomic polarizability is element specific, different *C*_3_ coefficients have to be used for different atomic species in the molecule. This is realized by the combination of element-specific *γ*_*i*_ and a single *C*_3_ which is, by our definition, the *C*_3_ for carbon. We cannot determine the ratios 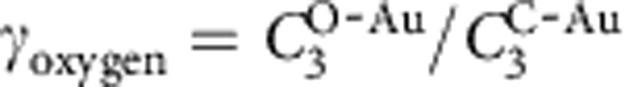 and 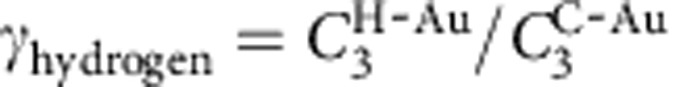 from our experimental data, because the fit quality *s* defined in equation (8) depends only very weakly on these ratios. The ratios are therefore taken from our vdW^surf^ calculation^11^, where *C*_3_ coefficients are calculated according to equation (6) with *ε*_S_(*iu*) coming from reflection energy-loss data^36^ and *α*_A*i*_(*iu*) from the polarizability of free atoms, scaled by an effective volume for the atom in the molecule that is determined by a Hirshfeld analysis^37^. The ratios *γ*_*i*_ are used to calculate the effective number of carbon atoms on the abscissa in Fig. 4a as


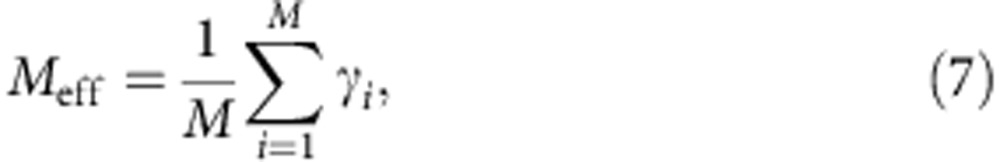


where *M* is the number of atoms in the molecule. We would like to point out that the observation of a non-additivity of the vdW potentials does not depend on the partitioning scheme used. For example, if we apply a uniform partitioning scheme with identical *C*_3_ for all atomic species, we obtain *C*_3_=18.7, *C*_3_=19.7 and *C*_3_=21.5 kcal mol^−1^ Å^3^ for NTCDA, PTCDA and TTCDA, respectively.

### Weighted least-squares regression

We use a weighted least-squares regression, minimizing the quantity


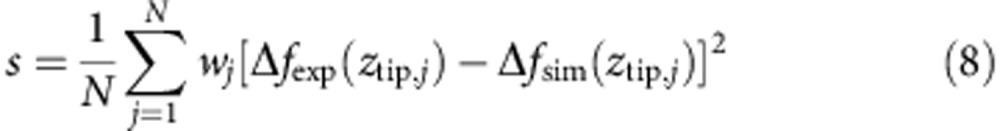


when fitting the *N* data points within the fit interval *j*=1,…*N*. Choosing the right weights *w*_*j*_ is a non-trivial task.

In the case of a linear fit, the goodness of fit (gof) is best described by the reduced *χ*^2^, defined as


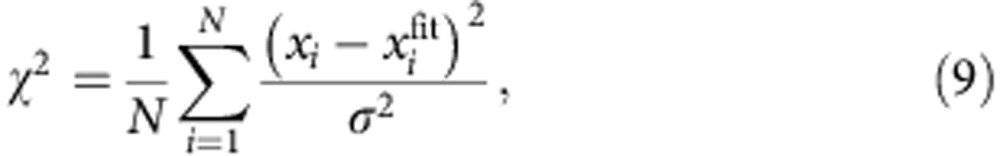


where *x*_*i*_ is the *i*th measured point and 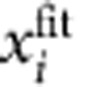 the corresponding value of the fitted curve. Here the respective weighting function is the inverse of the variance *σ*^2^ that is a measure of the statistical noise in the measured curve. While with this definition one obtains *χ*^2^=1 for a perfect fit, there is no general rule of how close a reduced *χ*^2^ should be to 1 for a good fit.

For a linear fit, the reduced *χ*^2^ as defined above is a suitable gof criterion, because according to this definition, each data point of a linear data set has the same chance of contributing to the overall *χ*^2^ value. However, in our case, we do not perform a linear fit. Rather, we fit a force law that is proportional to *z*^−5^ and moreover our experimental noise is practically constant over the whole fit interval. To gauge the quality of our fit to experimental data following this force law, we must ensure that the entire measured curve contributes to our gof criterion. Otherwise, experimental information would be lost, because any part of the measured data curve that does not contribute significantly to the gof criterion has no influence on the outcome of the fit. For this reason, we use the gof criterion in equation (8) with the weighting function


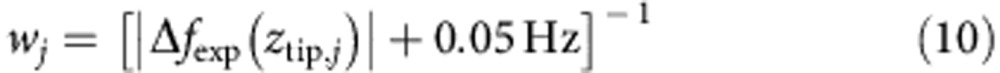


The normalization by the moduli of the data points ensures that differences Δ*f*_exp_−Δ*f*_sim_ that are small only because the measured value Δ*f*_exp_ and its fitted value Δ*f*_sim_ are close to zero, will nevertheless contribute to the overall gof *s*. The 0.05 Hz offset prevents singularities that can appear when the signal-to-noise ratio drops below unity and some data points come very close to zero. The value of 0.05 Hz is derived from the experimental noise level. However, fit results do not change significantly if, for example, 0.1 Hz is chosen instead.

A comparison of the two gof criteria *s* and *χ*^2^, displayed in Supplementary Fig. 6 clearly shows the advantage of our gof criterion *s* for fitting the *z*^−5^ power law. In the figure, we plot how the gof values *s* and *χ*^2^ accumulate as we sum over all *N* data points in the fit interval, starting close to the sample. The reduced *χ*^2^ criterion (blue) puts all the emphasis on the short-distance region (left side), while our gof function *s* (red) distributes the weight evenly across the whole data range.

The gof values *s* for our fit are displayed in Fig. 3. Having determined the best fit with the gof criterion *s*, we can also calculate a reduced *χ*^2^ value for our best fit. The respective value is *χ*^2^=2.3 (allowing different *C*_3_ for the three molecules). This is to be compared with a reduced *χ*^2^ value of 40 for the best fit that is obtained if the *C*_3_ coefficients of all molecules are constrained to be the same. The residuals of both fits are shown in Fig. 3.

### Fitting procedure

Because we expect similar reference plane positions *z*_0_ for all three molecules, only one *z*_0_ parameter is necessary for the three experimental Δ*f*(*z*_tip_) curves. Hence, the data of all three molecules are fitted simultaneously, minimizing a combined *s*. The parameters to be optimized in the fit are therefore *z*_0_, *C*_3,N_, *C*_3,P_, *C*_3,T_. In addition, a small absolute Δ*f* offset of each of the three experimental Δ*f*(*z*_tip_) curves is optimized. This offset is in the range of ±0.03 Hz. It accounts for a small remaining uncertainty in our data that is related to the approach curves which have the full ±0.4 Hz peak-to-peak noise level of a single non-averaged measurement. Although we eliminate this noise completely by using a fit to the approach curve instead of the approach curve itself, the fit is subject to a small uncertainty in said range of 0.03 Hz. To obtain a fully consistent picture, we correct this error by optimizing the respective offset during the fit. The experimental data shown in Fig. 3a have fitted offset values of 0.013 Hz (NTCDA), 0.02 Hz (PTCDA) and 0.0 Hz (TTCDA). As we do not presuppose a theoretical value for *z*_0_, but obtain it from the fit, any error in the molecule-surface distance determination would just result in a wrong value for *z*_0_, but not for the *C*_3_ coefficients (see, for example, equations (1)–(3), 4, 6). The fact that we obtain a value for *z*_0_ that is very close to theoretical expectations proves in turn that our initial distance determination is accurate within a small fraction of an Angstrom.

Since there may be local minima in the fit quality *s* as a function of the seven fit parameters, we use a robust fitting method that searches the whole parameter space around the expected minimum for *z*_0_ and the Δ*f* offset values, while using a method with decreasing step size for the optimization of the individual *C*_3_ coefficients.

### Experimental error via synthetic noise

An unavoidable source of error in the recovered *C*_3_ coefficients is the statistical noise in the experimental Δ*f* curves. It is not *a priori* clear how strongly the noise of about 0.02 Hz (NTCDA and TTCDA) and 0.05 Hz (PTCDA) affects our fitting procedure and thus the recovered *C*_3_ values. To estimate the error, we use a Monte Carlo approach, adding white noise of the respective amplitude to the data in Fig. 3a and conducting the fit. From the statistical distribution of the *C*_3_ values recovered in 140 such runs, we obtain an estimate of the statistical error. The resulting error bars are shown in Fig. 4a.

## Author contributions

R.T., F.S.T. and C.W. conceived the experiment. C.W., A.T. and F.S.T. conceived the concept of data analysis and interpretation. N.F. and R.T. conducted the experiments. C.W. performed the data analysis. V.G.R. and A.T. performed the DFT+vdW^surf^ calculations. M.R. performed the RPA calculations. C.L. and K.M. synthesized the TTCDA molecules. C.W. and F.S.T. wrote the manuscript, with significant contributions from A.T., M.R. and R.T.

## Additional information

**How to cite this article:** Wagner, C. *et al*. Non-additivity of molecule-surface van der Waals potentials from force measurements. *Nat. Commun.* 5:5568 doi: 10.1038/ncomms6568 (2014).

## Supplementary Material

Supplementary Information Supplementary Figures 1-9, Supplementary Table 1, Supplementary Discussion, Supplementary Methods and Supplementary References
