# Supplementary material for: Non-additivity of molecule-surface van der Waals potentials from force measurements
Source: Nat Commun. 2014 Nov 26;5:5568. doi: 10.1038/ncomms6568 (PMC4263323; doi:10.1038/ncomms6568)
Supplement: Supplementary Information — Supplementary Figures 1-9, Supplementary Table 1, Supplementary Discussion, Supplementary Methods and Supplementary References [file ncomms6568-s1.pdf]

1 Supplementary Figures

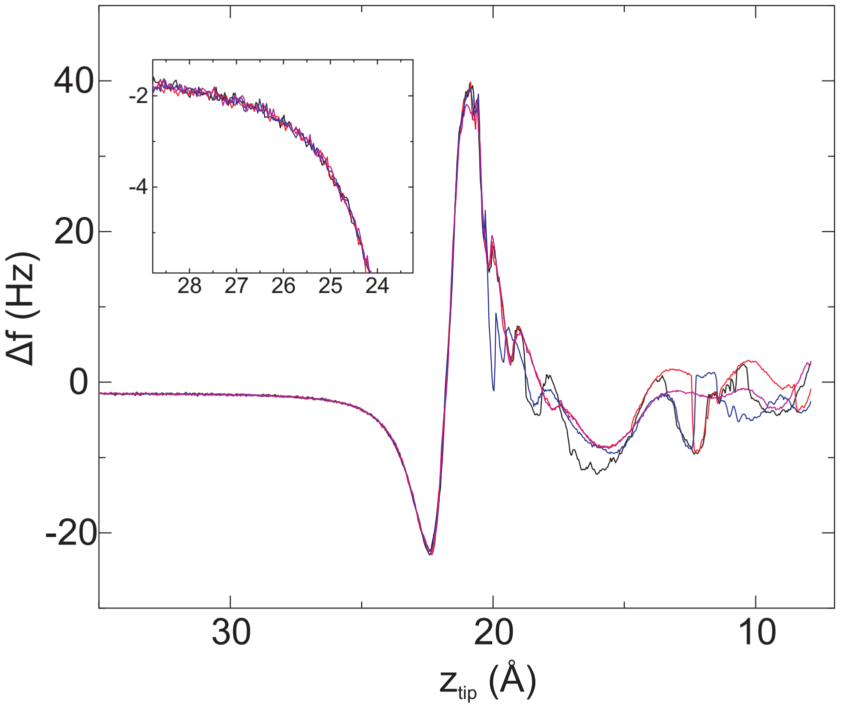

Supplementary Figure 1: **Exemplary regular raw data curves.** Displayed are four raw data curves taken during two consecutive tip cycles while lifting and lowering TTCDA. The inset shows the noise level and the reproducibility in a relevant region of the curve. The offset of about $1.8\,\mathrm{Hz}$ originates from the tip-sample interaction that is still present in the raw data.

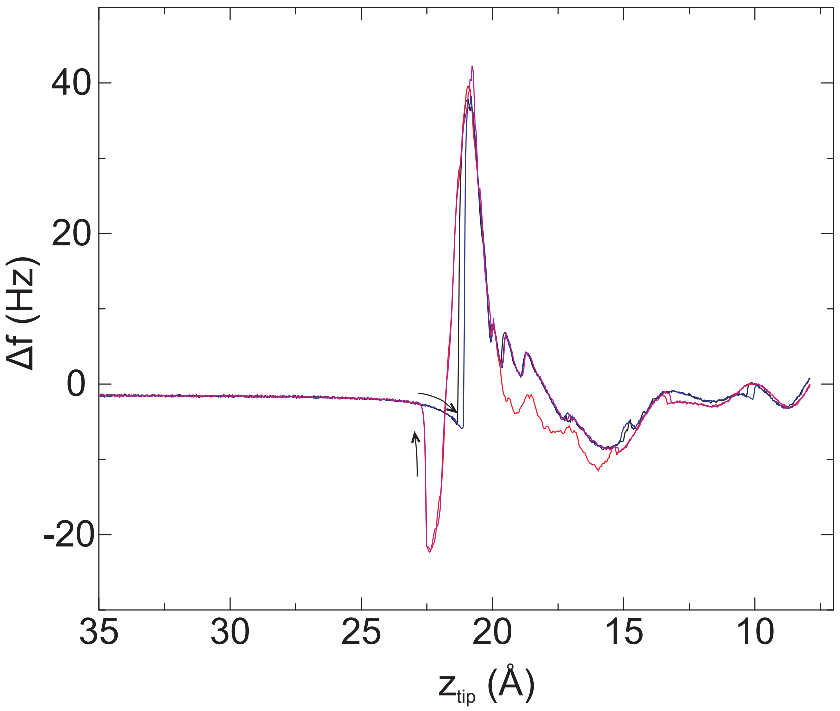

Supplementary Figure 2: **Exemplary irregular raw data curves.** Displayed are four raw data curves taken during two consecutive tip cycles while lifting and lowering TTCDA. The curves show a clear hysteresis between lifting and lowering of the molecules that originates from instability of the tip-suspended TTCDA molecule or of the tip.

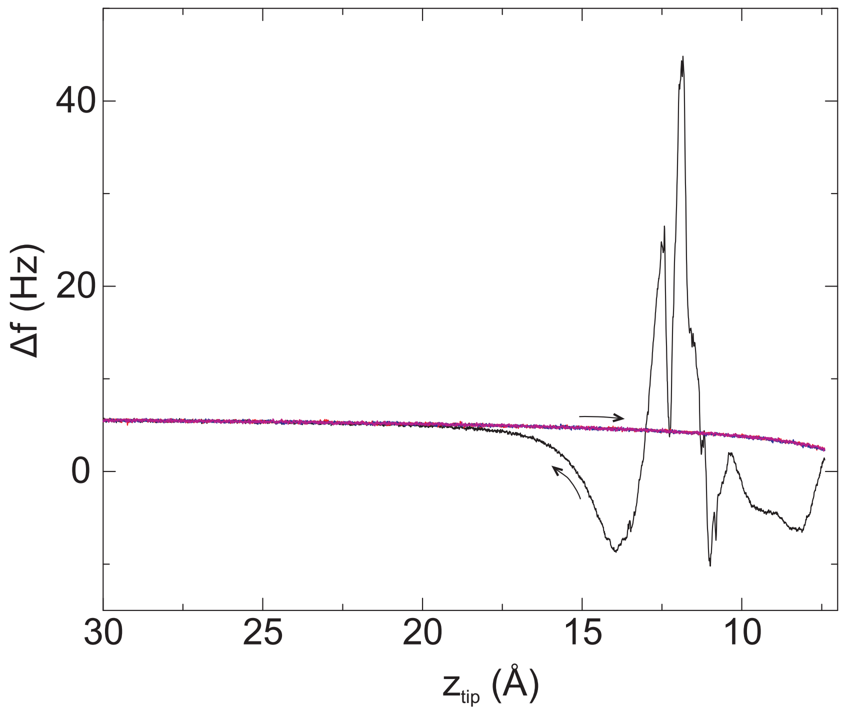

Supplementary Figure 3: **Exemplary regular and irregular raw data curves.** Displayed are four raw data curves taken during two consecutive tip cycles while lifting and lowering NTCDA. The molecule has flipped to the tip in the last part of the first tip retraction. The subsequently recorded curves show no sign of the molecule in the junction anymore.

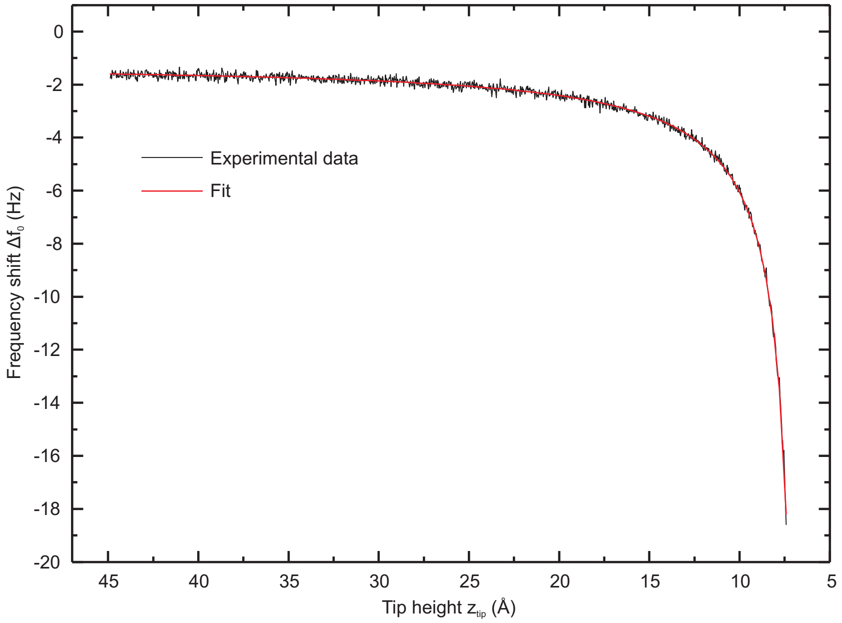

Supplementary Figure 4: **Exemplary bare tip approach curve and fit.** The fit to the approach curve is made separately for each contacting experiment. It is then subtracted from all Δf curves that have been measured within the respective contacting experiment. In this way we completely eliminate the contribution from the tip-surface interaction without introducing additional noise (from the approach curve) in the data.

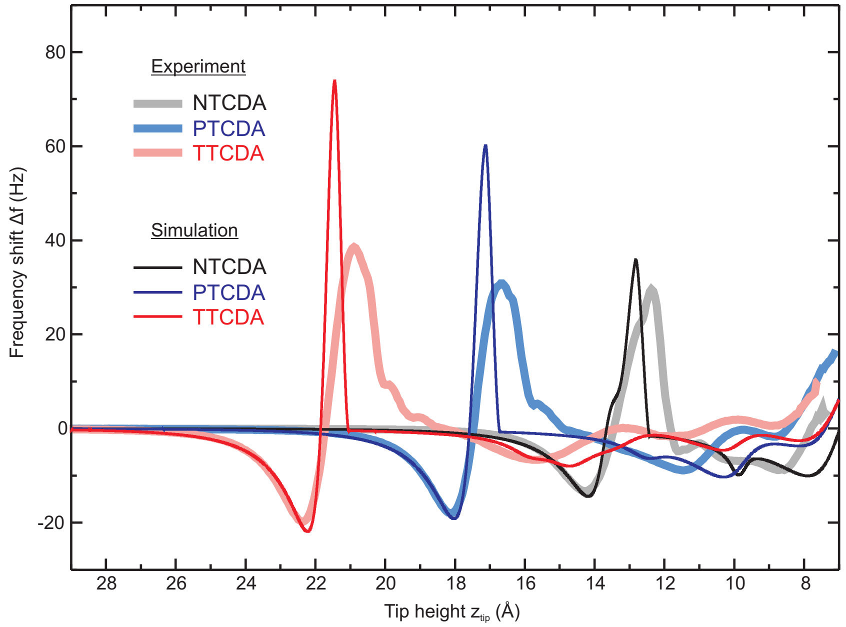

Supplementary Figure 5: **Determination of the absolute experimental z_{tip}-scale.** Experimental and simulated $\Delta f(z_{\text{tip}})$ curves for the complete single-molecule manipulation process for all three molecules. The experimental data has been aligned to the simulations[1] in the part of the curve where molecule and surface are well separated.

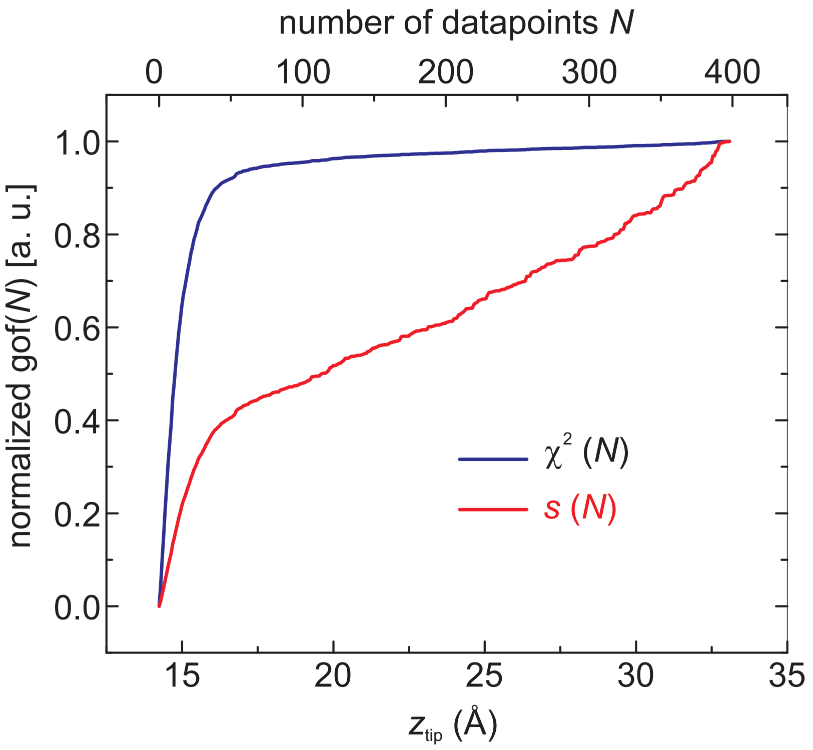

Supplementary Figure 6: **Comparison of the goodness of fit criteria χ^2 and *s*.** The exemplary gof values that are shown here have been calculated for a fit to the TTCDA data with a C_3 value which is $1\,\mathrm{kcal/mol\AA^3}$ higher than the optimal fit value. The normalized plots show how χ^2 and s add up to the sum in Eq. 8. The χ^2 criterion reaches 90% of its final value already after the first 10% of the fit interval, while the criterion s takes the full fit interval into account.

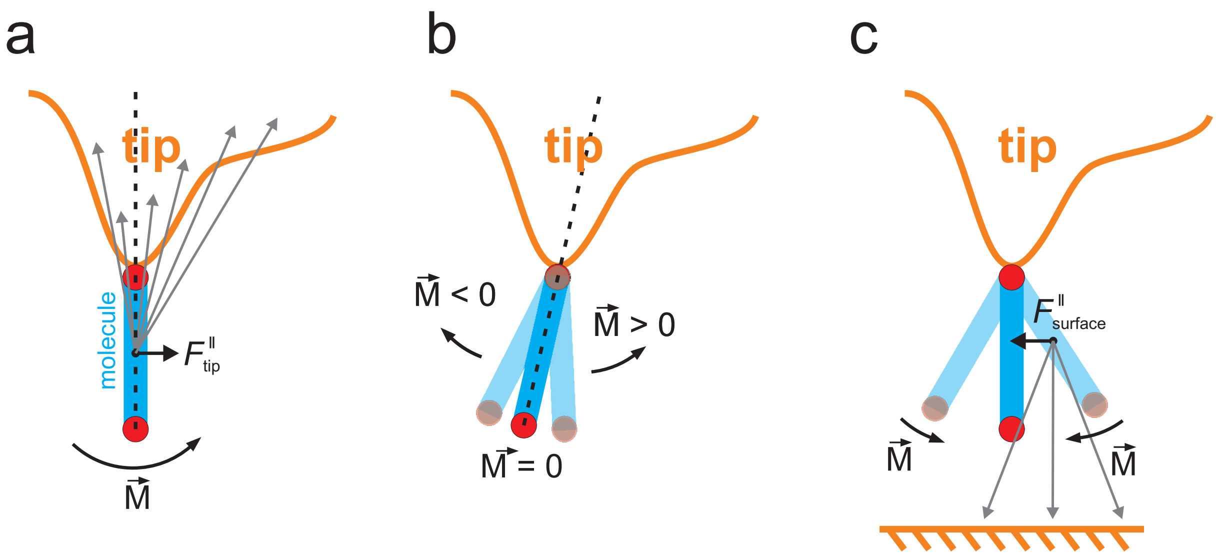

Supplementary Figure 7: **Sketch of the forces and resulting torques acting on the molecule.** **a** Asymmetries in the mesoscopic tip apex can lead to lateral forces acting on the tip-suspended molecule. **b** If the bond between molecule and tip apex has no directionality (as in the present case), and if the molecule experiences attractive forces to the tip only, the molecule will always end up sitting flat on the tip, because the equilibrium of a molecule not sitting flat on the tip will always be unstable. **c** The molecule-surface attraction introduces a restoring force strictly towards the vertical molecular orientation.

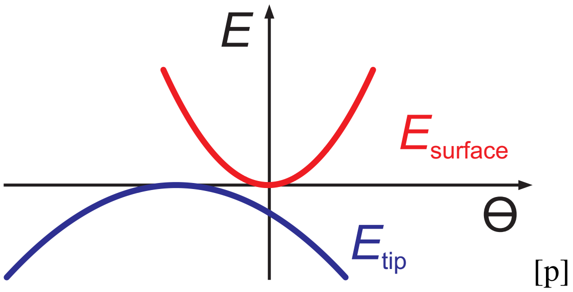

Supplementary Figure 8: **Schematic angle dependent potential.** Sketch of the potential energy of the molecule in Supplementary Figure 7

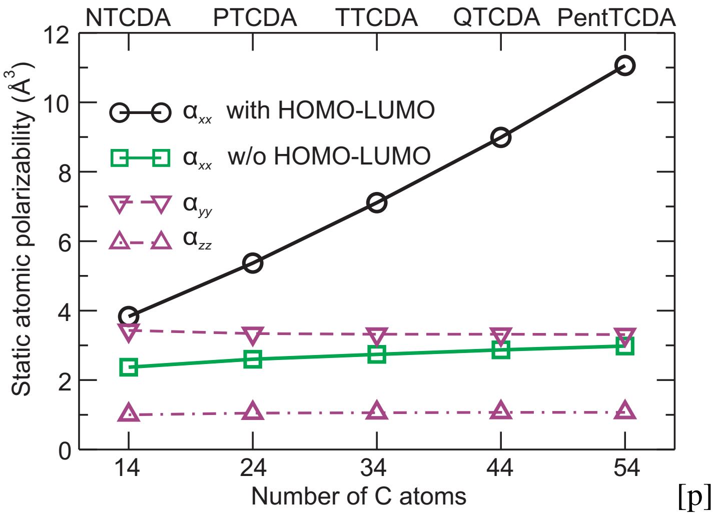

Supplementary Figure 9: **Static polarizabilities.** Static per-atom polarizabilities of carbon (averaged over all atoms in the molecule) for NTCDA, PTCDA, TTCDA, QTCDA, and PentTCDA (same as the zero-frequency data of panel a). The data labelled ”w/o HOMO-LUMO” are obtained by excluding the HOMO-LUMO transition from the summation of Supplementary Equation 6.

2 Supplementary Tables

	$C_3^{\mathrm{C}_m\mathrm{Au}}$	$C_3^{\mathrm{H}_m\mathrm{Au}}$	$C_3^{\mathrm{O}_m\mathrm{Au}}$	γ_{H}	γ_{O}	γ_{C}
NTCDA	27.0	7.7	18.0			
PTCDA	27.3	7.9	18.4	0.29	0.67	1.00
TTCDA	27.3	8.0	18.3			
Free atom	32.6	12.2	19.4	0.37	0.60	1.00

Supplementary Table 1: Interaction coefficients C_3 given in $\left(\mathrm{kcal\,mol^{-1}\,\AA^3}\right)$ for Au with each atomic species in the molecule and the weighting factors γ_{A} (dimensionless) are presented. The interaction coefficients of Au with each species A as a free atom are included for reference. By definition, γ_{C} is equal to 1.00.

3 Supplementary Discussion

Discussion and exclusion of potential systematic errors

qPlus sensor stiffness The stiffness of commercially available qPlus sensors is $1800 \pm 100\,\mathrm{N/m}$. This uncertainty introduces a systematic error in the determined C_3 coefficients of about 6%, which does, however, not influence the observed superlinearity.

Electrostatic forces In principle, electrostatic forces between the tip (with the attached molecule) and the surface could add to the experimentally measured frequency shifts and thus invalidate the fitting of our data that is based on van der Waals forces only. Electrostatic forces could originate from contact potential differences between the tip with attached molecule and the surface. Such contact potential differences could arise from charge transfer between the tip and the molecule. In particular, if there was a systematic variation in this charge transfer with the molecules in our homologuous series, electrostatic forces could influence the observed superlinearity of the C_3 coefficients. It is important to realize that we only need to consider electrostatic effects that are related to the tip-attached molecule, since *all* (bare) tip-surface interactions (van der Waals *and* electrostatic) are eliminated from the measured data by subtraction of the bare tip approach curve (see Methods section).

To address this issue, we have carried out DFT calculations of the tip-molecule-surface junction, with and without van der Waals interactions (PBE and PBE + **vdW**^{surf}). DFT calculations determine the charge distribution in the junction, including possible charge rearrangements be-

tween the tip (modeled by a 20 atom pyramidal cluster) and the molecule, and on this basis yield the electrostatic interaction energy (electrostatic forces) between the molecule-decorated tip and the surface. We note here that the DFT calculations are very expensive, because the system-size is large and the sought-after interaction energies are very small, on the border of what is feasible.

Our DFT calculations show that there is no systematic change of the charge transfer between tip and molecule in the homologous series NTCDA-PTCDA-TTCDA, and hence no systematic change of the contact potential that could explain the superlinearity of our C_3 coefficients. In fact, the (molecule related; see above) electrostatic interaction energies between the molecule-decorated tips and the surface are exceedingly small (at $z_{\mathrm{mol}} = 7\,\mathrm{Å}$ $-3.1\,\mathrm{meV}$ for NTCDA, $-5.1\,\mathrm{meV}$ for PTCDA and $-2.1\,\mathrm{meV}$ for TTCDA) and, most importantly, do not increase in the sequence from NTCDA to TTCDA, as would be necessary if systematic contact potential differences were to be invoked to explain the superlinearity of the experimentally determined C_3 coefficients. Moreover, even at the relatively close distance of $z_{\mathrm{mol}} = 7\,\mathrm{Å}$, the DFT+vdW^{surf} calculated van der Waals interaction energies are approximately one order of magnitude larger (26.6 to 33.5 meV) than the electrostatic energies. Furthermore, there is strong experimental evidence (confirmed by DFT calculations) that also for PTCDA on a flat Au(111) surface there is essentially no charge transfer to the molecule[12]. Since no charge transfer is present in either of the two extreme cases (sharp Au tip and flat Au(111) surface), we can conclude that charge transfer for our real experimental tip is also negligible.

On the basis of the detailed DFT study, we can thus conclude that electrostatic interaction

does not play a significant role in the present material system xTCDA/Au(111). This is in full agreement with our experimental findings and the interpretation of our data.

Orientation of the molecule In our fitting of equation (2) to the experimental data we employ a vertical orientation of the molecule (i.e. the diagonal of the molecule is perpendicular to the surface). This orientation is obtained by minimizing the molecule-surface vdW potential for the tip-suspended molecule. If there was in experiment a deviation from this orientation, this would have an influence on the determined C_3 coefficients (this is, in fact, obvious from equation 2). However, we demonstrate below by means of an ab-initio DFT calculation and experimental evidence that obtaining the molecular orientation by minimizing the molecule-surface vdW potential (and thus the vertical orientation as defined above) is indeed justified.

In our DFT calculation (PBE functional) we start with a molecule that is suspended vertically off the tip and tilt this molecule in the molecular plane and perpendicular to it. The incurred energy cost is of the order of $10\,\mathrm{meV}$ in an angle range of $\pm 20°$ (the tilting perpendicular to the molecular plane incurs an even smaller energy cost of $< 2\,\mathrm{meV}$). Since these numbers are at the significance threshold of DFT, the calculation confirms that for our molecules the gold-oxygen bond has essentially no directionality. The orientation of the molecule hanging off the tip will therefore be determined by van der Waals forces alone (electrostatic forces are negligible, as discussed above). There are two sets of van der Waals forces of relevance here: (1) F_{surface} between the molecule and the surface and (2) F_{tip} between the molecule and the tip. For symmetry reasons, F_{surface} is always directed strictly normal to the surface, while F_{tip} can (and most probably

will) deviate from this direction, depending on asymmetries of the tip shape (see Supplementary Figure 7a). $F_{\rm tip}^{||}$, the parallel component of $F_{\rm tip}$, is related to the imbalance between the attractions to opposite sides of the tip and produces a torque M on the molecule, as indicated in Supplementary Figure 7a.

If for the moment we disregard $F_{\rm surface}$, we see in Supplementary Figure 7b that on an asymmetric tip the molecule will assume an equilibrium position ($M = 0$) that is tilted away from the more massive side of the tip. In this way the presence of more tip matter on the right hand side is balanced by the closer vicinity of the molecule to the left hand side of the tip (Supplementary Figure 7b). What is also immediately obvious from Supplementary Figure 7b is the fact that the equilibrium of a molecule that experiences only attractive forces to the tip is always unstable: Any tilt to one side will produce a torque that will turn the molecule further to that same side, until the molecule sits flat on the tip. This is shown schematically by the curve marked with $E_{\rm tip}$ in Supplementary Figure 8, where the potential energy E is plotted against the tilt angle θ. We can thus conclude: If the bond between molecule and tip apex has no directionality (as in the present case), and if the molecule experiences attractive forces to the tip only, the molecule will always end up sitting flat on the tip. Evidently, such a molecule is undetectable in our experiments (see Supplementary Figure 3).

The facts that (i) in many cases we still detect the molecule in the junction when we return the tip towards the surface from far away, and that (ii) the corresponding frequency shift curves fall on top of the Δf curves measured on retraction of the tip with the hanging molecule from the

surface (i.e. no hysteresis), thus mean that there must be additional forces acting on the molecule which turn the unstable equilibrium of the hanging molecule into a stable one, such that no flip to the tip can occur. These forces can only originate from the surface (since the tip-molecule bond is not directional, see above). Indeed, Supplementary Figure 7c shows that van der Waals attraction to the surface generates torques that turn the molecule towards the vertical orientation. The corresponding potential energy curve is also shown in Supplementary Figure 8, labelled E_{surface}. The total potential energy is given by the sum of the two curves (not drawn).

Now Supplementary Figure 8 shows very clearly: In order for the molecule to survive the whole manipulation cycle in the hanging configuration (with maximum tip-surface distances of 45 Å at the upper turning point), the total potential energy curve must be opening upwards throughout the complete cycle (otherwise the molecule would flip to the tip). Therefore the potential curve E_{tip} must always be shallower than E_{surface} in this cycle. Since the van der Waals attraction to the surface at the upper turning point of the manipulation cycle is very weak and therefore E_{surface} is very shallow, the potential energy curve E_{tip} must be even shallower. Note that E_{tip} itself does not depend on the tip-surface distance. In other words, the restoring force constant $k^{||}_{\text{surface}}$ due to the minimal van der Waals attraction to the surface constitute an upper bound for the parallel force constant $k^{||}_{\text{tip}}$ due to the tip throughout the complete manipulation cycle (i.e. $k^{||}_{\text{surface}} > k^{||}_{\text{tip}}$ always, with $k^{||}_{\text{surface}} >> k^{||}_{\text{tip}}$ for most of the manipulation cycle since $F_{\text{surface}} \propto z^{-4}_{\text{mol}}$ increases rapidly with decreasing z_{mol}).

In conclusion, this means that in all those manipulation cycles in which the molecule survives

in the hanging configuration, the molecule must reside very close to the vertical configuration for most of the fitting range, because in most the fitting range $k^{||}_{\mathrm{surface}} >> k^{||}_{\mathrm{tip}}$. We note here that the improvement of the fit due to allowing for superlinearity is achieved in a distance range quite close to the sample (see Fig. 3a of the manuscript) where the surface-derived torque on the molecule is strong, leading to a vertical orientation. Hence, we can positively exclude that our finding of superlinearity is influenced in any way by a deviation of the molecular orientation from the vertical.

According to Supplementary Figures. 7a and 7b, a tip with a small $k^{||}_{\mathrm{tip}}$ is a nearly symmetric tip. One may ask why we have succeeded in preparing a relatively large number of symmetric tips. The answer is clear: Only symmetric tips allow complete, non-hysteretic manipulation cycles. By eliminating hysteric curves form our data set, we automatically eliminate asymmetric tips which would lead to significantly tilted molecules. This means in summary: While it is true that tilts of the molecule will influence the obtained C_3 values, we can exclude such tilts on experimental grounds and thus obtain C_3 coefficients that are not influenced by tilts.

Deviations from the asymptotic dispersion interaction at short range

We found in the paper that below ≈ 4.8 Å the asymptotic form Eq. 5 of the dispersion interaction is not fulfilled any more (Fig. 3b). This is to be expected, because Eqs. 5 and 6 only constitute the long-range limit of the more general expression for the interaction part ($V_{\mathrm{a-s}}$) of the entire correlation energy E_{C} (see Methods section). In the random phase approximation (RPA) to the adiabatic-connection fluctuation-dissipation theorem (ACFDT)[15,17–19] the correlation energy E_{C} is

given by

$$E_{\rm C} = -\frac{1}{2\pi}\int_0^\infty du \mathrm{Tr}\left(\underline{\underline{1}} - \underline{\underline{\epsilon}}(iu) + \ln(\underline{\underline{\epsilon}}(iu))\right) \qquad (1)$$

with $\underline{\underline{\epsilon}}(\omega)$ being the space-dependent dielectric function (or, equivalently, its Fourier-transform matrix as discussed above in the Supplementary Methods) of the entire system of surface plus atom/molecule.

The RPA total energy, being an approximation, might suffer from slight inaccuracies on an absolute energy scale. Nonetheless, RPA is perfectly suitable as a basis on which the deviation of van der Waals attraction from its long-range asymptotic behaviour at close distances can be discussed.

At large distances the charge densities of substrate and atom/molecule do not overlap, and $\underline{\underline{\epsilon}}(\omega)$ can be simplified into individual contributions from surface and atom/adsorbate, finally leading to the expressions of Eqs. 5 and 6. At closer distances, however, the charge densities and polarizabilities of surface and of atom/molecule overlap and influence each other in a way which prohibits separation. The evaluation of Supplementary Equation 1 for physisorption of PTCDA on Ag(111) shows that $V_{\rm a-s}(z)$ becomes weaker than Eq. 5 for z smaller than about 4-5 Å [15], and does not diverge at z_0. This makes the use of Eq. 5 questionable at typical physisorption distances, as is indeed found in our fits if the fitting region starts at too low values of $z_{\rm mol}$.

Superlinearity

In the paper we found a clear trend of increasing experimental C_3 coefficients with molecular size, i.e. the per-atom molecule-surface interaction rises in the sequence NTCDA $\rightarrow$ PTCDA $\rightarrow$ TTCDA. Slight inaccuracies of the RPA total energy on an absolute energy scale notwithstanding, the RPA (see discussion above and Supplementary Methods) is perfectly suitable as a basis to discuss the connection between the microscopic electronic states and the macroscopic polarizability of the molecule that finally leads to the superlinear increase of the C_3 coefficients.

Supplementary Equations 6 to 10 indicate two important issues regarding the macroscopic polarizability α_{macr} and its separation into contributions from individual atoms. On the one hand, the microscopic charge-density response χ in Supplementary Equation 6 can often be discussed in terms of local, atom-wise polarizability effects (simply because the various wave functions have local contributions at the various atoms). However, after the transformation $\chi \rightarrow \epsilon \rightarrow \epsilon^{-1} \rightarrow \epsilon^{-1}_{\mathrm{macr}} \rightarrow \alpha_{\mathrm{macr}}$, which shuffles information around in real space (in terms of the so-called local-field effects), atom-wise additivity is questionable. This is in principal contrast to the idea of the additivity of atom-to-atom contributions to the van der Waals interaction. Fortunately, such effects are often of secondary importance. In the present case of NTCDA, PTCDA, TTCDA and beyond, our calculations indicate that the molecular polarizabilities before and after the $\chi \rightarrow \epsilon \rightarrow \epsilon^{-1} \rightarrow \epsilon^{-1}_{\mathrm{macr}} \rightarrow \alpha_{\mathrm{macr}}$ transformation differ by a factor that is the same for all molecules discussed here. This would re-establish the additivity, if it were not for the second issue to which we now turn.

Supplementary Equation 6 indicates that even at the level of the microscopic density response

function χ, the polarizability of an atom is drastically influenced by its environment, because the latter affects the wave functions and the energy levels. This influence can be quite long-ranged if delocalized quantum mechanical states dominate the polarizability. We discuss this issue in more detail below for the case of NTCDA, PTCDA, and TTCDA. In short, the delocalized nature of quantum mechanical states makes it impossible to accurately predict the polarizability of an atom from just considering its nearest neighbours.

As an example, we show in Supplementary Figure 9a the per-atom polarizability of the carbon atoms (averaged over all carbon atoms in each molecule) for NTCDA, PTCDA, and TTCDA for imaginary frequencies iu that are relevant for Eq. 6 and Supplementary Equation 1. The data result from Supplementary Equation 6 by partitioning the wave functions into contributions at each atom (for details, see Ref. 21). We also evaluate the anisotropy of the charge-density response of Supplementary Equation 6, which is particularly important for the current case of flat π-conjugated systems. Note that all discussion here are on the level of χ (more precisely $(\underline{\underline{\chi}}(\omega))_{\mathbf{Q}=\mathbf{0},\mathbf{Q}'=\mathbf{0}}$), i.e. Supplementary Equation 6, without considering local-field effects.

Three important observations can be made in Supplementary Figure 9a:

- At large iu, the per-atom polarizability behaves as $\chi \propto u^{-2}$. This is expected from Supplementary Equation 6 if $u \gg E_n - E_m$. Moreover, at large iu the per-atom polarizability is essentially the same for all molecules.
- There is a strong anisotropy of the per-atom polarizability. This is coupled to the linear ex-

tensions of the molecule in the three space directions. The larger the extension in a given direction, the larger the respective polarizability. Note that NTCDA, which is of similar extension in x and y directions, also has similar per-atom polarizabilities in these two directions.

- As $iu \to 0$, we observe a strong difference in χ_{xx} between NTCDA, PTCDA, and TTCDA. Looking at Supplementary Equation 6, we would expect such a behaviour if $E_n - E_m$ was decreasing in the same sequence. Since the HOMO-LUMO transition is by definition the lowest energy transition in each molecule that therefore contributes strongest to the atomic polarizability $\chi \propto (E_n - E_m)^{-1}$ in Supplementary Equation 6, this suggests that the HOMO-LUMO gap drops in the sequence NTCDA $\to$ PTCDA $\to$ TTCDA. This is indeed true. It is remarkable that χ_{yy} and χ_{zz} are apparently not affected by the changing HOMO-LUMO gap, with the consequence that the per-atom polarizabilities along y and z are nearly the same for all molecules. This is also found for all per atom polarizabilities of the oxygen and hydrogen atoms.

The strong increase of the per-atom polarizability along x (the long axis of the molecule) for increasing length of the molecule (NTCDA$\to$PTCDA$\to$TTCDA) is the origin of the superlinearity of the C_3 coefficients as discussed in the paper.

A more detailed analysis confirms the role of the HOMO-LUMO transition as conjectured above. In Supplementary Figure 9b the same data as in panel **a** is displayed, but for zero frequency only (static case). For the data labelled ”w/o HOMO-LUMO” the HOMO-LUMO transition has

been left out of the summation in Supplementary Equation 6. Evidently, the per-atom polarizability along the x axis is strongly affected, in particular for the larger molecules where this transition dominates the polarizability in x direction. Without this transition only a very small superlinearity persists (from 2.37 Å^3 to 2.98 Å^3 when going from NTCDA to PentTCDA). This shows that more than 90 % of the superlinearity results from the HOMO-LUMO transition of the molecules.

Due to the particular symmetry of the HOMO and LUMO wave functions, the polarizabilities in the y and z directions are not affected at all by the HOMO-LUMO transition. Apparently, this transition shuffles π electrons exclusively in the direction of the long axis of the molecule. In this context, it is interesting to note that in going from the HOMO to the LUMO, the number of nodal planes perpendicular to the long axis of the molecule increases from 5 to 6, with the consequence that the superposition of these two orbitals can shift charge along the long molecular axis effectively, as required for a polarization in this direction.

For an infinitely long molecule, i.e. a $(C_{10}H_4)_n$ polymer, electrons would form a metallic band and the static polarizability in x direction would diverge. Our five data points of Supplementary Figure 9b constitute the onset of this divergence. For finite molecules, the band breaks up into confined states, which are the molecular orbitals[22], e.g. HOMO and LUMO. The longer the molecule, the more closely spaced are these confined states on the energy axis, i.e., the HOMO-LUMO gap reduces from NTCDA to PentTCDA. According to Supplementary Equation 6 ($\chi \propto (E_n - E_m)^{-1}$), this leads to increasing contributions of corresponding electronic transitions to the low-frequency molecular polarizability. Note that the C_3 coefficients follow this trend, but

they would not diverge at infinite molecular size.

It is well-known that the conjugation in aromatic systems can lead to a superlinear increase of the polarizability with molecular size, most prominently in elongated species such as oligomers[23–25]. The influence of this effect on the optical properties of molecules in solution is subject of intense research[26,27]. Here, we observe superlinearity for the first time directly in force experiments. One has to keep in mind that the superlinear rise of the dispersion interaction is smaller than the one in optical experiments, because the latter probe the polarizability at optical frequencies, while the dispersion potential is obtained by integration over imaginary frequencies up to $100\,\mathrm{eV}$.

Full potential profile between PTCDA and Au(111)

Fig. 4b contains two separate interaction potentials for PTCDA with Au(111), one for the near-distance region, the other for the asymptotic region. Between them, they fully map out the potential profile.

The asymptotic potential is based on the data of this paper. In Fig. 4b we follow common practice and plot the potential for the flat molecule (molecular plane parallel to the surface). Since for a flat molecule all atoms in the molecule have identical z, we can sum over the C_3 coefficients of all atoms in the molecule. The total potential thus becomes

$$V_{\mathrm{flat\,mol-s}} = -M_{\mathrm{eff}} \frac{C_3}{(z-z_0)^3}, \tag{2}$$

with the effective number of carbon atoms M_{eff} (Eq. 7).

The orange potential profile is based on $C_3 = 70\,\mathrm{kcal/mol\AA^3}$ with $z_0 = 0\,\mathrm{\AA}$, as determined in Ref. 1 by fitting the range from 8 to 14.7Å and from 16.9 to 23.6Å in Fig. 2a (blue curve) with an expression based on the asymptotic potential Eq. 5. It is clear that applying this expression outside its range of validity, z_0 will decrease while C_3 will increase when approaching the surface, thus emulating the more shallow slope of the true potential for $z < 4.8\,\mathrm{\AA}$ and avoiding the singularity at z_0 (see Ref. 15 and Supplementary Discussion above). This behaviour can clearly be seen in Fig. 3b. We note here that although the $C_3 = 70\,\mathrm{kcal/mol\AA^3}$ and $z_0 = 0\,\mathrm{\AA}$ are 'unphysical' in the sense that they have been derived outside the asymptotic region, they do parameterize the true potential sufficiently well to yield a reasonable adsorption energy[1]. For clarity, the repulsive branch of the short-range potential (Ref. 1) has been omitted in Fig. 4b.

4 Supplementary Methods

The DFT+vdW$^{\rm surf}$ methodology

The DFT+vdW$^{\rm surf}$ method consists in the combination of the DFT+vdW method[2] for treating intermolecular interactions in density-functional theory (DFT) and the Lifshitz-Zaremba-Kohn (LZK) theory[3,4] for the nonlocal many-body response of the substrate surface. The inclusion of these collective many-body effects, present within the substrate, in the determination of the vdW interaction effectively goes beyond an atom-based pairwise description.

Based on the LZK theory, the interaction coefficient $C_3^{\mathrm{A}_m\mathrm{S}}$ between atomic species A_m and substrate S is given by

$$C_3^{\mathrm{A_m S}} = \frac{\hbar}{4\pi}\int_0^\infty d\omega \frac{\varepsilon_{\mathrm{S}}(i\omega)-1}{\varepsilon_{\mathrm{S}}(i\omega)+1}\,\alpha_{\mathrm{A}_m}(i\omega), \tag{3}$$

where $\epsilon_{\mathrm{S}}(i\omega)$ is the dielectric function of solid S (Au in the present case) calculated from reflection energy–loss spectroscopy experiments[5] and $\alpha_{\mathrm{A}_m}(i\omega)$ corresponds to the dipole polarizability of atomic species A in molecule m, where $m = \{\mathrm{N}, \mathrm{P}, \mathrm{T}\}$ as defined in the main text for [N]TCDA, [P]TCDA, and [T]TCDA. For the element-specific dipole polarizabilities $\alpha_{\mathrm{A}_m}(i\omega)$, we rely on a Padé approximant model[2,6] as in the DFT+vdW method, given by the leading term of the Padé series as:

$$\alpha_{\mathrm{A}_m}(i\omega) = \frac{\alpha^0_{\mathrm{A}_m}[n(\mathbf{r})]}{1+(\omega/\eta_{\mathrm{eff}})}, \tag{4}$$

where η_{eff} is an effective characteristic excitation frequency and $\alpha^0_{\mathrm{A}_m}(i\omega)$ corresponds to an element-specific isotropic static dipole polarizability. The latter is a functional of the ground-state electron

density $n(\mathbf{r})$ of the atom in the molecule given as $\alpha^0_{\mathrm{A}_m}[n(\mathbf{r})] = v^{\mathrm{A}_m}_{\mathrm{eff}}[n(\mathbf{r})]\alpha^0_{\mathrm{A}_{\mathrm{free}}}$, where $\alpha^0_{\mathrm{A}_{\mathrm{free}}}$ corresponds to the static dipole polarizability of the free atom obtained from high level quantum chemical calculations, and $v^{\mathrm{A}_m}_{\mathrm{eff}}[n(\mathbf{r})]$ is the definition of a dimensionless effective volume for species A_m referenced to the free atom in terms of the Hirshfeld partitioning of the electron density (see Refs. 2, 7–9).

In order to compute the effective volumes $v^{\mathrm{A}_m}_{\mathrm{eff}}$ for each species A in molecule m, we performed DFT calculations for the case of a single [N,P,T]TCDA molecule on a Au(111) surface using the PBE exchange-correlation functional[10]. The effective volumes are given by

$$v^{\mathrm{A}_m}_{\mathrm{eff}} = \left(\frac{\int r^3 w_{\mathrm{A}}(\mathbf{r}) n^m(\mathbf{r}) d^3\mathbf{r}}{\int r^3 n^{\mathrm{free}}_{\mathrm{A}}(\mathbf{r}) d^3\mathbf{r}} \right), \tag{5}$$

where $w_{\mathrm{A}}(\mathbf{r})$ is the Hirshfeld atomic partitioning weight of the species A, r^3 is the cube of the distance from the nucleus of an atom A, $n^m(\mathbf{r})$ is the total electron density of molecule m, and $n^{\mathrm{free}}_{\mathrm{A}}(\mathbf{r})$ is the reference electron density corresponding to the free atom A.

All calculations were performed with the FHI-AIMS code[11]. The repeated-slab method was used to model all three systems, with three metallic layers to perform the calculations in-line with previous investigations[7,12–15]. We used the experimental lattice constant of Au (4.065 Å) to build the (111) surface and a Monkhorst-Pack[16] grid of $2 \times 2 \times 1$ k-points in the reciprocal space. For each system, the volumes $v^{\mathrm{A}_m}_{\mathrm{eff}}$ were calculated with the molecule placed at distances larger than 4.0 Å with respect to the topmost unrelaxed substrate layer. The final values taken for the calculation of $\alpha^0_{\mathrm{A}_m}(i\omega)$ correspond to the distance averaged value in each system.

Using the theory described above, we computed the interaction coefficient $C_3^{\mathrm{A}_m\mathrm{Au}}$ for C, H, and O in the [N,P,T]TCDA molecules with Au(111) as substrate. We define the weighting factor γ_{A} for species A with respect to Carbon as $\gamma_{\mathrm{A}} = \frac{1}{3}\sum_m^3 \gamma_{\mathrm{A}_m}$, where $\gamma_{\mathrm{A}_m} = C_3^{\mathrm{A}_m\mathrm{Au}}/C_3^{\mathrm{C}_m\mathrm{Au}}$ and $m = \{\mathrm{N}, \mathrm{P}, \mathrm{T}\}$ as described in the text above. The results are given in Supplementary Table 2.

C_3 coefficients from the random phase approximation

Fig. 4a contains C_3 coefficients based on the random phase approximation (RPA) to the adiabatic-connection fluctuation-dissipation theorem (ACFDT)[15,17–19]. The calculation is based on Eq. 6 with RPA-calculated molecular polarizabilities and ϵ_{S} for the metal from Ref. 20.

Since we are in the limit of clearly separated subsystems surface and molecule, we have to use the *macroscopic* dipole polarizability α_{macr} which is defined as the dipole response to the *macroscopic* electric field. But we need to calculate the macroscopic response of the molecule from a full *microscopic* quantum theory of the molecule, because we are interested in the role played by the quantum mechanical electronic states in the response.

The evaluation of the α in Eq. 6 starts with the microscopic density-density response function

$$\chi(\mathbf{r}, \mathbf{r}', \omega) = -4\frac{1}{V}\sum_{m}^{occ}\sum_{n}^{empty} \psi_m(\mathbf{r})\psi_m^*(\mathbf{r}')\psi_n^*(\mathbf{r})\psi_n(\mathbf{r}')\frac{E_n - E_m}{(E_n - E_m)^2 - \omega^2} \qquad (6)$$

at the RPA level. This function reveals how a perturbation of the density at position $\mathbf{r}$ within the molecule propagates to position $\mathbf{r}'$. The $|m\rangle$, $|n\rangle$ are the quantum mechanical states of the electrons in the molecule. From Supplementary Equation 6, we calculate the Fourier transform $(\underline{\underline{\chi}}(\omega))_{\mathbf{Q},\mathbf{Q}'}$, where in our notation the matrix $(\underline{\underline{f}})_{\mathbf{Q},\mathbf{Q}'}$ is the Fourier transform of $f(\mathbf{r}, \mathbf{r}')$. $(\underline{\underline{\chi}}(\omega))_{\mathbf{Q},\mathbf{Q}'}$ is related

to the microscopic dielectric function $(\underline{\underline{\epsilon}}(\omega))_{\mathbf{Q},\mathbf{Q}'}$ by a matrix-matrix multiplication with the bare Coulomb interaction $(\underline{\underline{v}}(\omega))_{\mathbf{Q},\mathbf{Q}'}$

$$(\underline{\underline{\epsilon}}(\omega))_{\mathbf{Q},\mathbf{Q}'} = 1 - (\underline{\underline{v}})_{\mathbf{Q},\mathbf{Q}'}(\underline{\underline{\chi}}(\omega))_{\mathbf{Q},\mathbf{Q}'} \tag{7}$$

In Fourier space, we can obtain the inverse microscopic dielectric function $(\underline{\underline{\epsilon}}^{-1}(\omega))_{\mathbf{Q},\mathbf{Q}'}$ by a simple matrix inversion. The *macroscopic* dielectric constant is defined as the long-range limit of the inverse dielectric function, which can be obtained in Fourier space as

$$\epsilon_{\mathrm{macr}}^{-1}(\omega) = (\underline{\underline{\epsilon}}^{-1}(\omega))_{\mathbf{Q}=0,\mathbf{Q}'=0}. \tag{8}$$

Finally, we have to relate the macroscopic dielectric function to the macroscopic polarizability that can be used in Eq. 6. Here we use the fact that the polarization density $\mathbf{P} = \mathbf{p}/\mathbf{V}$, where V is the volume, can be expressed by

$$\mathbf{P}(\mathbf{r}) = \frac{\epsilon_{\mathrm{macr}} - \mathbf{1}}{\mathbf{4}\pi}\mathbf{E}(\mathbf{r}), \tag{9}$$

where $\mathbf{E}(\mathbf{r})$ is the macroscopic electric field. Combining this with the definition of the macroscopic polarizability $\mathbf{p}(\mathbf{r}) = \alpha_{\mathrm{macr}}\mathbf{E}(\mathbf{r})$ we obtain

$$\alpha_{\mathrm{macr}}(\omega) = \frac{V}{4\pi}(\epsilon_{\mathrm{macr}}(\omega) - 1). \tag{10}$$

$\alpha_{\mathrm{macr}}(\omega)$ at imaginary frequencies is then inserted into Eq. 6. We note that for anisotropic objects like the molecules under consideration here, different directions along which the $\mathbf{Q}, \mathbf{Q}'$ approach zero in Supplementary Equation 8 lead to different dielectric functions. In other words, $\epsilon_{\mathrm{macr}}(\omega)$ and $\alpha_{\mathrm{macr}}(\omega)$ are 3×3 tensors. Because of the symmetry of our molecules, these tensors become diagonal if the coordinate system is chosen to coincide with the three symmetry directions (principal axes), such that the macroscopic polarizability is fully described by the three functions $\alpha_{xx}(\omega)$,

$\alpha_{yy}(\omega)$ and $\alpha_{zz}(\omega)$. Note that we use a molecular coordinate system in which x refers to the long molecular axis within the plane of the molecule, y refers to the short axis, and z is perpendicular to the molecular plane.

5 Supplementary References

1. Wagner, C., Fournier, N., Tautz, F. S. & Temirov, R. Measurement of the binding energies of the organic-metal perylene-teracarboxylic-dianhydride/Au(111) bonds by molecular manipulation using an atomic force microscope. *Phys. Rev. Lett.* **109**, 076102 (2012).

2. Tkatchenko, A. & Scheffler, M. Accurate molecular van der Waals interactions from ground-state electron density and free-atom reference data. *Phys. Rev. Lett.* **102**, 073005 (2009).

3. Lifshitz, E. M. The theory of molecular attractive forces between solids. *Soviet Phys. JETP* **2**, 73–83 (1956).

4. Zaremba, E. & Kohn, W. Van der Waals interaction between an atom and a solid surface. *Phys. Rev. B* **13**, 2270 (1976).

5. Werner, W. S. M., Glantschnig, K. & Ambrosch-Draxl, C. Optical constants and inelastic electron-scattering data for 17 elemental metals. *J. Phys. Chem. Ref. Data* **38**, 1013 (2009).

6. Tang, K. T. & Karplus, M. Padé-approximant calculation of the nonretarded van der Waals coefficients for two and three helium atoms. *Phys. Rev.* **171**, 70 (1968).

7. Ruiz, V. G., Liu, W., Zojer, E., Scheffler, M. & Tkatchenko, A. Density-functional theory with screened van der Waals interactions for the modeling of hybrid inorganic-organic systems. *Phys. Rev. Lett.* **108**, 146103 (2012).

8. Hirshfeld, F. L. Bonded-atom fragments for describing molecular charge densities. *Theor. Chim. Acta* **44**, 129 (1977).

9. Johnson, E. R. & Becke, A. D. A post-Hartree-Fock model of intermolecular interactions. *J. Chem. Phys.* **123**, 024101 (2005).

10. Perdew, J., Burke, K. & Ernzerhof, M. Generalized gradient approximation made simple. *Phys. Rev. Lett.* **77**, 3865 (1996).

11. Blum, V. *et al.* Ab initio molecular simulations with numeric atom-centered orbitals. *Comput. Phys. Commun.* **180**, 2175 (2009).

12. Romaner, L., Nabok, D., Puschnig, P., Zojer, E. & Ambrosch-Draxl, C. Theoretical study of PTCDA adsorbed on the coinage metal surfaces, Ag(111), Au(111) and Cu(111). *New J. Phys.* **11**, 053010 (2009).

13. Hauschild, A. *et al.* Molecular distortions and chemical bonding of a large π-conjugated molecule on a metal surface. *Phys. Rev. Lett.* **94**, 036106 (2005).

14. Rohlfing, M., Temirov, R. & Tautz, F. S. Adsorption structure and scanning tunneling data of a prototype organic-inorganic interface: PTCDA on Ag(111). *Phys. Rev B* **76**, 115421 (2007).

15. Rohlfing, M. & Bredow, T. Binding energy of adsorbates on a noble-metal surface: Exchange and correlation effects. *Phys. Rev. Lett.* **101**, 266106 (2008).

16. Monkhorst, H. J. & Pack, J. D. Special points for brillouin-zone integrations. *Phys. Rev. B* **13**, 5188 (1976).

17. Furche, F. Molecular tests of the random phase approximation to the exchange-correlation energy functional. *Phys. Rev B* **64**, 195120 (2001).

18. Marini, A., Garcia-Gonzalez, P. & Rubio, A. First-principles description of correlation effects in layered materials. *Phys. Rev. Lett.* **96**, 136404 (2006).

19. Harl, J. & Kresse, G. Cohesive energy curves for noble gas solids calculated by adiabatic connection fluctuation-dissipation theory. *Phys. Rev. B* **77**, 045136 (2008).

20. Lynch, D. & Hunter, W. *Handbook of Optical Constants of Solids*, 286 (Academic Press, Boston, 1985).

21. Rohlfing, M. Electronic excitations from a perturbative LDA+GdW approach. *Phys. Rev. B* **82**, 205127 (2010).

22. Koller, G. *et al.* Intra- and intermolecular band dispersion in an organic crystal. *Science* **317**, 351–355 (2007).

23. Zhao, M.-T., Singh, B. P. & Prasad, P. N. A systematic study of polarizability and microscopic third-order optical nonlinearity in thiophene oligomers. *J. Chem. Phys.* **89**, 5535–5541 (1988).

24. Thienpont, H., Rikken, G. L. J. A. & Meijer, E. W. Saturation of the hyperpolarizability of oligothiophenes. *Phys. Rev. Lett.* **65**, 2141 (1990).

25. Samuel, I. D. W. *et al.* Saturation of cubic optical nonlinearity in long-chain polyene oligomers. *Science* **265**, 1070 (1994).

26. Tretiak, S. & Mukamel, S. Density matrix analysis and simulation of electronic excitations in conjugated and aggregated molecules. *Chem. Rev.* **102**, 3171 (2002).

27. Kuzyk, M. G., Pérez-Moreno, J. & Shafei, S. Sum rules and scaling in nonlinear optics. *Phys. Rep.* **529**, 297 (2013).
